# A Wide-Ranging Antiviral Response in Wild Boar Cells Is Triggered by Non-coding Synthetic RNAs From the Foot-and-Mouth Disease Virus Genome

**DOI:** 10.3389/fvets.2020.00495

**Published:** 2020-08-04

**Authors:** Miguel Rodríguez Pulido, Ranjitha H. B., Margarita Sáiz

**Affiliations:** Centro de Biología Molecular Severo Ochoa, CSIC-UAM, Madrid, Spain

**Keywords:** foot-and-mouth-disease virus, antivirals, wild boar, non-coding RNA, wildlife

## Abstract

Foot-and-mouth disease virus (FMDV) is the causative agent of a highly contagious viral disease that affects multiple cloven-hooved hosts including important livestock (pigs, cattle, sheep and goats) as well as several wild animal species. Crossover of FMDV between domestic and wildlife populations may prolong virus circulation during outbreaks. The wild boar (*Sus scrofa*) is considered a reservoir of various pathogens that can infect other wildlife, domestic animals, and humans. As wild boar and domestic pigs are susceptible to the same pathogens and can infect each other, infected wild boar populations may represent a threat to the pig industry and to international trade. The ncRNAs are synthetic non-coding RNA transcripts, mimicking structural domains in the FMDV genome, known to exert a broad-spectrum antiviral and immunomodulatory effect in swine, bovine and mice cells. Here, we show the type I interferon-dependent, robust and broad range antiviral activity induced by the ncRNAs in a cell line derived from wild boar lung cells (WSL). Transfection of WSL cells with the ncRNAs exerted a protective effect against infection with FMDV, vesicular stomatitis virus (VSV), swine vesicular disease virus (SVDV) and African swine fever virus (ASFV). Our results prove the biological activity of the ncRNAs in cells of an FMDV wild animal host species against a variety of viruses affecting pigs, including relevant viral pathogens of epizootic risk.

## Introduction

Foot-and-mouth disease (FMD) is a severe, highly contagious and transboundary viral disease that has a significant economic impact affecting the production of livestock and disrupting regional and international trade in animals and animal products. The causative agent of FMD is foot-and-mouth disease virus (FMDV), a member of the family *Picornaviridae*. FMDV isolates are classified into seven different serotypes and all of them have been found in wildlife (1). The capacity of the wild boar (*Sus scrofa*) for FMDV transmission has been reported and the prolonged viral secretion along with mild clinical disease raised the concern that wild boars may spread FMD (2, 3). However, our knowledge on the clinical manifestations of FMD in wild boars and their actual contribution to transmission during field outbreaks is very limited (4–6). Wild boars are extremely adaptable, presenting a current geographic range that comprises territories from three continents. The Eurasian wild boar is widely distributed in Europe and hunting bags reveal a massive increase in the population in recent decades. This population growth may lead to increased contact with the domestic pig, consequently increasing the risk of transmission of pathogens (7, 8). As a result, infected wild boar populations may represent a threat to the pig industry and to international trade. How this affects the risk of FMD in Europe is a relevant aspect to be considered (5).

Here, we have assayed the antiviral activity in wild boar cells of three synthetic non-coding RNA molecules derived from the FMDV genome (ncRNAs) against FMDV and other relevant viral pathogens of domestic swine. The ncRNAs mimic in sequence and structure the 5′-terminal S fragment (S), the internal ribosome entry site (IRES) and the 3′ non-coding region (3′NCR), respectively (9, 10). These small and non-infectious RNA transcripts are known to elicit a robust antiviral effect based on type I interferon (IFN) induction through both Toll-like and retinoic acid-inducible gene-I (RIG-I)-like receptors (TLR and RLR, respectively) signaling pathways (11–14). The IRF3-dependent activation of the antiviral responses triggered by the 3′NCR transcripts in swine and bovine cells has been described (15). The FMDV S fragment has also been involved in modulation of innate responses in host cells (16). In previous work, we showed the enhancing effect of the IRES transcripts on the specific B- and T-cell mediated immune responses elicited by a conventional inactivated FMD vaccine in pigs, increasing the rate of protection against FMDV challenge (17). With the aim of testing the biological activity and potential application of these immunomodulatory RNA molecules in FMDV wild host species, the immune response and antiviral spectrum of the ncRNAs has been analyzed in a wild boar cell line (WSL). Our results show that transfection of wild boar cells with the ncRNAs triggered a solid and broad range innate immune response. The antiviral activity induced in transfected WSL cells effectively inhibited infection by FMDV and also by three other relevant viruses: vesicular stomatitis virus (VSV) and swine vesicular disease virus (SVDV)—two RNA viruses causing vesicular disease in pigs—and moreover, by African swine fever virus (ASFV), a complex DNA viral pathogen causing a highly virulent disease of domestic swine with devastating consequences for swine industries and food security globally.

## Materials and Methods

### Cells and Viruses

WSL cell line was developed in Günther Keil laboratory (Friedrich-Loeffler-Institut, Greifswald, Germany) from wild boar lung cells (18). WSL cells were shown to have a macrophage lineage origin with the loss of some specific myeloid markers (19). Vero cells were obtained from ATCC. Swine kidney epithelial IBRS2 cells were obtained from CISA-INIA. WSL, Vero, and IBRS2 cells were grown in Dulbecco's modified Eagle's medium supplemented with 10% fetal calf serum, 100 μg/ml penicillin-streptomycin and 2 mM L-glutamine (Gibco). FMDV O1BFS isolate, vesicular stomatitis virus (VSV) Indiana, swine vesicular disease virus (SVDV) SPA 93 and African swine fever virus BA71 V9 (adapted to Vero cells) were used for infection experiments. Information of titers of FMDV, VSV, SVDV and ASFV viral stocks used in this study, as well as those in WSL cells is shown in Table 1.

**Table 1:** Viral titers of the viruses used in the study in WSL cells compared to those in the cell lines where viral stocks were grown.

**Virus**	**Titer in WSL**	**Titer of viral stock (cell line)**
FMDV	3 × 10^6^ pfu/ml	1 × 10^7^ pfu/ml (SK6)
VSV	4.9 × 10^8^ pfu/ml	2.9 × 10^9^ pfu/ml (BHK21)
SVDV	2 × 10^7^ pfu/ml^(a)^	3.7 × 10^7^ pfu/ml (IBRS2)
ASFV	2.4 × 10^6^ TCID_50_/ml^(b)^	1.4 × 10^6^ pfu/ml (Cos)

(a) *supernatants from SVDV-infected WSL cells were titered in IBRS2 cells*.

(b) *supernatants from ASFV-infected WSL cells were titered in Vero cells*.

### RNA Synthesis, Transfection, and RT-PCR

RNA *in vitro* transcripts corresponding to the 3′NCR (186 nt including a 58-nt polyA tail) or S fragment (5′-terminal 403 nt) of the FMDV O1K genome were synthetized using T3 RNA polymerase (NEB) and previously described plasmids as templates that were linearized with *Not*I prior to *in vitro* transcription (10). RNA corresponding to the IRES of FMDV CS8 (470 nt) was generated by *in vitro* transcription with T7 RNA polymerase (NEB) from a pGEM-derived clone (20) linearized with *Xho*I. Next, DNA was removed from the RNAs preparations by treatment with RQ1 DNase (1 U/μg; Promega). Then, RNAs were extracted with phenol-chloroform, ethanol-precipitated, and finally resuspended in water. The RNA was quantified by spectrometry and its size and integrity were analyzed by electrophoresis. RNAs were denatured/renatured by heating at 92°C for 5 min, incubation for 10 min at room temperature, and then kept on ice until transfection. In some experiments, *E. coli* MRE600 tRNA (Roche) and pI:C (Invivogen) were used as negative and positive controls, respectively. Transfection was performed using Lipofectamine 2000 (Invitrogen). Approximately 1 × 10^6^ WSL cells were transfected with 40 μg/ml 3′NCR, S, IRES transcripts or tRNA. For RT-PCR analysis, cells were harvested at different times following transfection. Then, total RNA was extracted, quantified by spectrometry and treated for DNA removal with Turbo DNA-free kit (Ambion). RNA aliquots were subsequently analyzed by RT-PCR for amplification of swine IFN-β and Myxovirus resistance gene 1 (Mx1) or GAPDH as described (14, 21). Amplification products were detected and analyzed by electrophoresis on agarose gels (2–2.5%).

### Antiviral Activity Assays

The paracrine antiviral activity of the supernatants from ncRNA-transfected WSL cells against VSV or FMDV was assayed on WSL cells, while the activity against SVDV was assayed on IBRS2 cells. The assays were performed basically as described (10). Briefly, WSL cells were transfected for 24 h with 40 μg/ml of the ncRNAs transcripts, pI:C (Invivogen), tRNA or mock-transfected with PBS. Fresh monolayers of WSL (or IBRS2 for SVDV) cells were incubated for 24 h with the transfection supernatants (serial dilutions), washed, and infected with 50–100 PFU/10^6^ cells (MOI of 0.5–1 × 10^−4^) of VSV, FMDV or SVDV. Next, the plaques were counted 24 h after infection with VSV and FMDV or 48 h after infection with SVDV, respectively. Where indicated, the blockade of the antiviral activity in the transfection supernatants was assessed by previous incubation of the supernatants with 2 μg of specific neutralizing monoclonal antibodies against swine IFN-α (K9; PBL InterferonSource) for 1 h at 37°C. Antiviral activity was expressed as the reciprocal of the highest dilution of the corresponding supernatant reducing the number of plaques by 50%.

A VSV infection inhibition assay was performed to assess the autocrine antiviral activity induced in WSL cells by transfection with the FMDV ncRNAs. For that, WSL cells were mock-transfected or transfected with 40 μg/ml of tRNA, S, IRES or 3′NCR transcripts and infected 24 h after transfection with 50–100 PFU/10^6^ cells (MOI of 0.5–1 × 10^−4^) of VSV. Cytophatic effect (CPE) was monitored by plaque assay on semi-solid medium 24 h after infection.

To test the autocrine antiviral effect of ncRNA transfection in WSL cells against ASFV, WSL cells were mock-transfected or transfected with IRES RNA as above, and 24 h later, infected with ASFV at an MOI of 2. Cell extracts were collected at 4, 8, or 16 h after infection. The viral titers were determined by plaque assay in Vero cells at 5 days post-infection and expressed as TCID50/ml.

### Immunoblot Analysis

Detection of total IRF3, phospho-IRF3 and Mx1 was performed by SDS-PAGE. IRES-transfected WSL cells were washed twice in ice-cold PBS and harvested in PBS supplemented with 1% NP-40, 1 mM DTT and 1X Complete protease inhibitor cocktail (Roche) at the indicated times after transfection. Cell extracts (20 μg) were run on 10% SDS-PAGE gels, transferred onto nitrocellulose membrane and probed with the specific primary antibody. Then, incubation of the blots with the corresponding secondary antibody HRP conjugate (Thermo Scientific Pierce) was performed. Proteins were detected by chemiluminescent detection (NZY standard ECL, NZYTech) followed by exposure to X-ray film. The following primary antibodies were used in this study: rabbit monoclonal anti-Phospho-IRF3 (Ser 396) (4D4G, Cell Signaling), rabbit polyclonal anti-IRF3 (FL-425, Santa Cruz Biotech), mouse monoclonal anti-Mx1 (AM39, Acris Antibodies) and rabbit polyclonal anti-βII tubulin (22).

### Statistical Analysis

For comparison of data, the unpaired Student's *t*-test for independent samples was used with the IBM SPSS Statistical (v.24) software; statistically significance was considered for a *p* < 0.05. As mentioned in the corresponding figure legends, ns indicates not significant (*p* > 0.05), ^*^p < 0.05, ^**^p < 0.01 and ^***^p < 0.001.

## Results and Discussion

### The FMDV IRES Triggers a Type I IFN-Dependent Innate Immune Response in Wild Boar Cells

**Figure 1:**
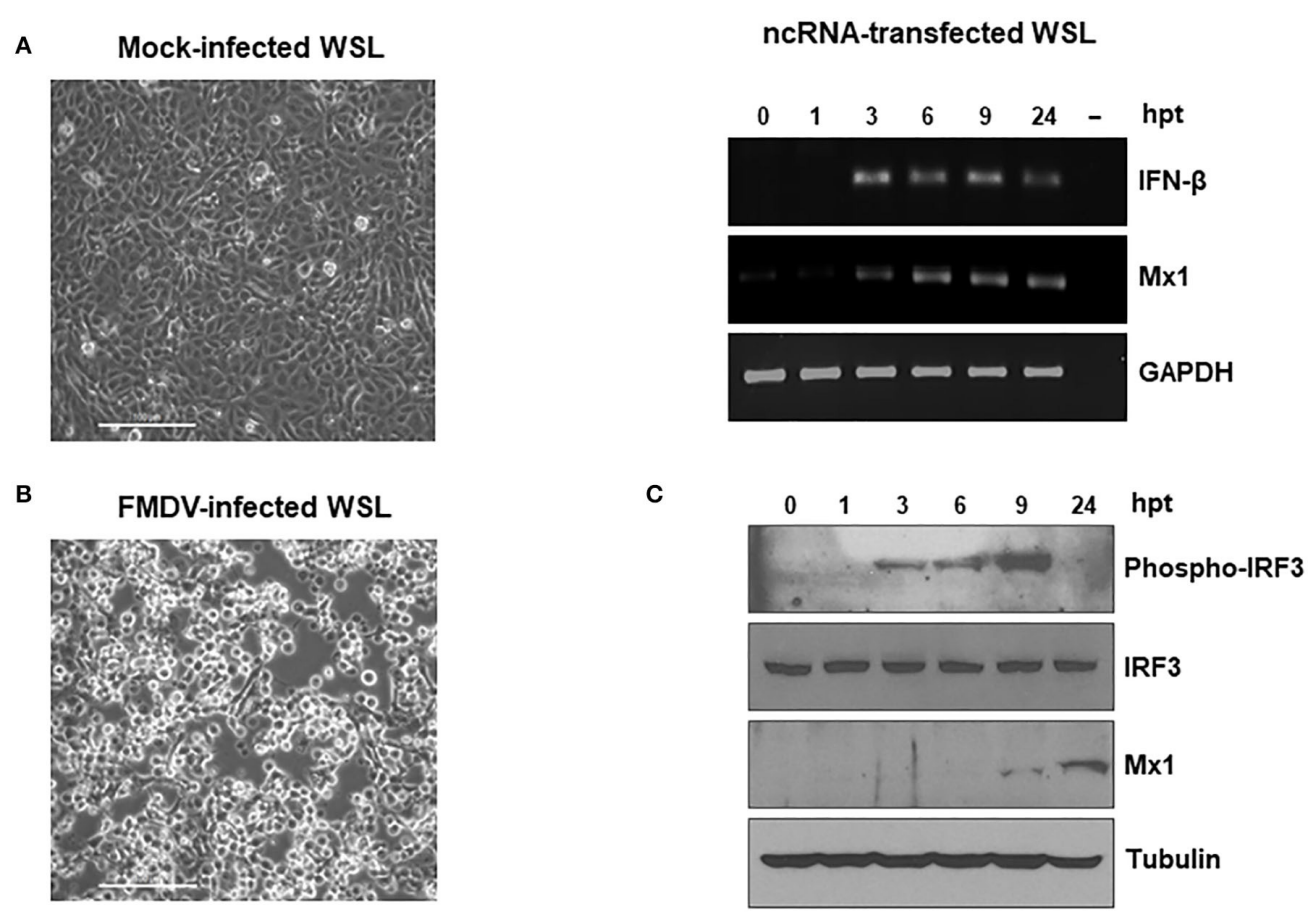
WSL cells are susceptible to FMDV infection and induce innate immune responses upon transfection with the ncRNAs. **(A)** WSL cells were infected with FMDV O1BFS at an MOI of 0.01 or mock-infected. Images were captured 24 h after infection. Scale bars, 100 μm. **(B,C)** WSL cells were transfected with IRES RNA and cell lysates were collected at the indicated times after transfection for RT-PCR analysis of IFN-β, Mx1 and GAPDH mRNAs **(B)** or for protein detection of phospho-IRF3, total IRF3, Mx1, and tubulin by immunoblot **(C)**.

To assess the potential protective effect of the ncRNAs in wild boar cells against FMDV infection, we first tested the susceptibility of WSL cells to the virus. When WSL were infected with FMDV, a clear CPE could be observed, being the extent of it dependent on the MOI used. Figure 1A shows the comparison between WSL monolayers 24 h after either infection with FMDV at an MOI of 0.01 or mock infection. Next, the effect of transfection with the IRES transcripts on IFN-β mRNA induction in WSL cells was analyzed. The 470 nt long RNA transcripts corresponding to the IRES in the 5′ NCR of the FMDV genome conferred the highest levels of protection against FMDV in mice (11) and were also able to enhance the immune response of an FMD vaccine in mice and pigs (17, 23). The RT-PCR analysis of WSL cells transfected with the IRES showed the induction of IFN-β mRNA, being detectable from 3 to 24 h after transfection (Figure 1B). The mRNA levels of Mx1 were also analyzed. *Mx1* is an IFN-stimulated gene (ISG) involved in anti-FMDV response in swine and bovine cells (24–28). Mx1 mRNA induction was also observed between 3 and 24 h after transfection with IRES transcripts in WSL cells (Figure 1B). We were also able to detect the expression of Mx1 protein at 9 and 24 h post-transfection, indicating that the gene induction observed led to productive translation of the protein (Figure 1C). As a result of viral infection and subsequent activation of the signaling routes (including TLR and RLR pathways) leading to promote an antiviral state, the cytoplasmic inactive form of IRF3 undergoes phosphorylation of a series of serine residues, dimerization and translocation to the nucleus where a protein complex is formed for activation of the type I IFN and ISG genes (29). To address whether IRF3 was being activated in ncRNA-transfected WSL cells, the levels of phosphorylated and total IRF3 in the lysates were analyzed by immunoblot (Figure 1C). While total IRF3 levels remained stable over time, phospho-IRF3 was initially detected at 3 h after transfection, reaching maximal levels at 9 h post-transfection, the later time coinciding with the initial detection of Mx1 protein (Figure 1C). Our results show that the FMDV ncRNAs can trigger type I IFN-dependent innate immune responses in wild boar cells.

### Transfection With the ncRNAs Confers Protection Against VSV, FMDV, SVDV, and ASFV Infection in WSL Cells

**Figure 2:**
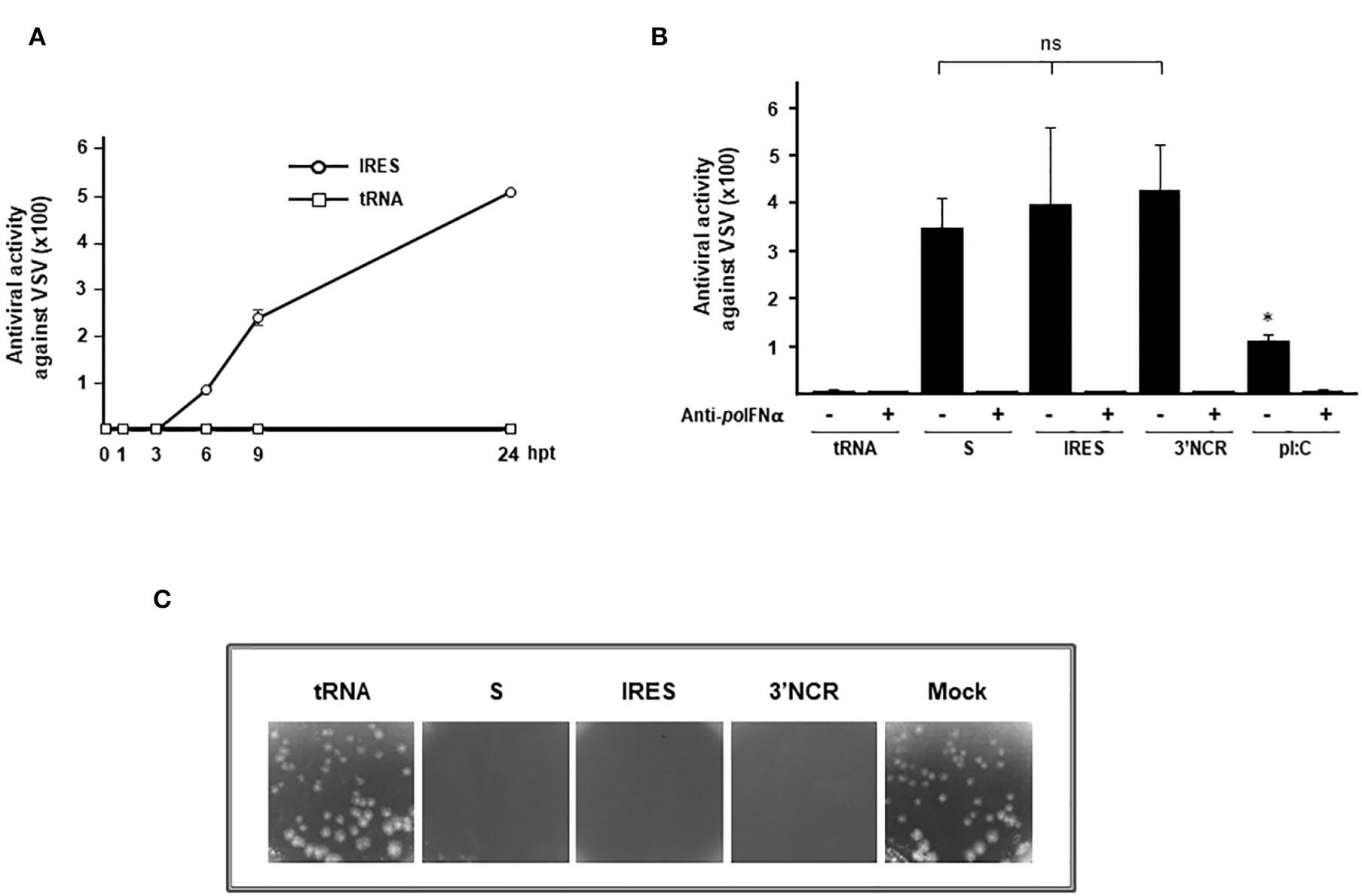
Antiviral activity against VSV induced in WSL cells by transfection with the ncRNAs. **(A)** Antiviral activity of supernatants from IRES-transfected WSL cells, collected at different times after transfection and corresponding to lysates analyzed in Figures 1B,C. Supernatants from WSL cells transfected with tRNA were also analyzed as a control. The antiviral activity was assayed on fresh WSL monolayers against VSV infection. Data are average of triplicates ± SD. **(B)** The antiviral activity in supernatants from WSL cells transfected with S, IRES, 3′NCR transcripts, pI:C or tRNA for 24 h was assayed on fresh WSL monolayers against VSV. Where indicated, supernatants were incubated previously with antibodies against swine IFN-α. Data are average of triplicates from two independent experiments ± SD (*p < 0.05; ns, not significant). Antiviral activity was expressed as the reciprocal of the highest supernatant dilution needed to reduce the number of VSV plaques by 50%. **(C)** Autocrine antiviral activity in WSL cells transfected with the ncRNAs. WSL cells were transfected with each ncRNA, tRNA or mock-transfected and 24 h later infected with VSV. A comparison of the CPE induced after 24 h of infection is shown.

Having shown the upregulation of IFN-β and ISGs in IRES-transfected WSL cells (Figures 1B,C) we sought to analyze whether the innate immune response elicited was associated with measurable antiviral activity. For that, we first tested the supernatants corresponding to IRES-transfected WSL for paracrine antiviral activity against VSV. As shown in Figure 2A, the antiviral activity increased over time being first detected at 6 h after transfection and reaching maximal levels 24 h post-transfection (around 500). No antiviral activity could be measured in supernatants from WSL cells transfected with tRNA (Figure 2A). Next, the paracrine antiviral activity in supernatants from WSL cells 24 h after transfection with each ncRNA or with pI:C (a double stranded RNA analog) was tested against VSV (Figure 2B). High levels of antiviral activity were found in all supernatants from ncRNA-transfected cells, with no statistically significant differences between S, IRES or 3′NCR RNAs. Transfection with pI:C induced 3–3.8-fold lower levels of antiviral activity than the FMDV ncRNAs. No sign of cytotoxicity or negative effect on cell viability was observed after transfection with any of the RNAs analyzed. In all cases, incubation with an anti-swine IFN-α antibody abrogated the antiviral activity in the supernatants (Figure 2B). This is in agreement with previous work showing that IFN-α mainly accounts for the antiviral activity in swine transfected or FMDV infected cells (10, 30) despite the early induction of IFN-β mRNA observed. A possible explanation for this may be that IFN-β is translated at lower levels or that its turnover rate mRNA/protein is very rapid (30). With the aim of testing the autocrine antiviral activity induced by the ncRNAs in wild boar cells, WSL monolayers were transfected with tRNA, S, IRES, 3′NCR RNAs, or mock-transfected and 24 h later, cells were infected with VSV. While the CPE observed in tRNA- and mock-transfected cells was equivalent, no sign of infection was detected in WSL cells transfected with each of the three ncRNAs, suggesting that transfected cells were protected against VSV infection (Figure 2C). Altogether, these results show that the FMDV ncRNAs are able to induce a fast and potent autocrine and paracrine antiviral response in wild boar cells against VSV.

**Figure 3:**
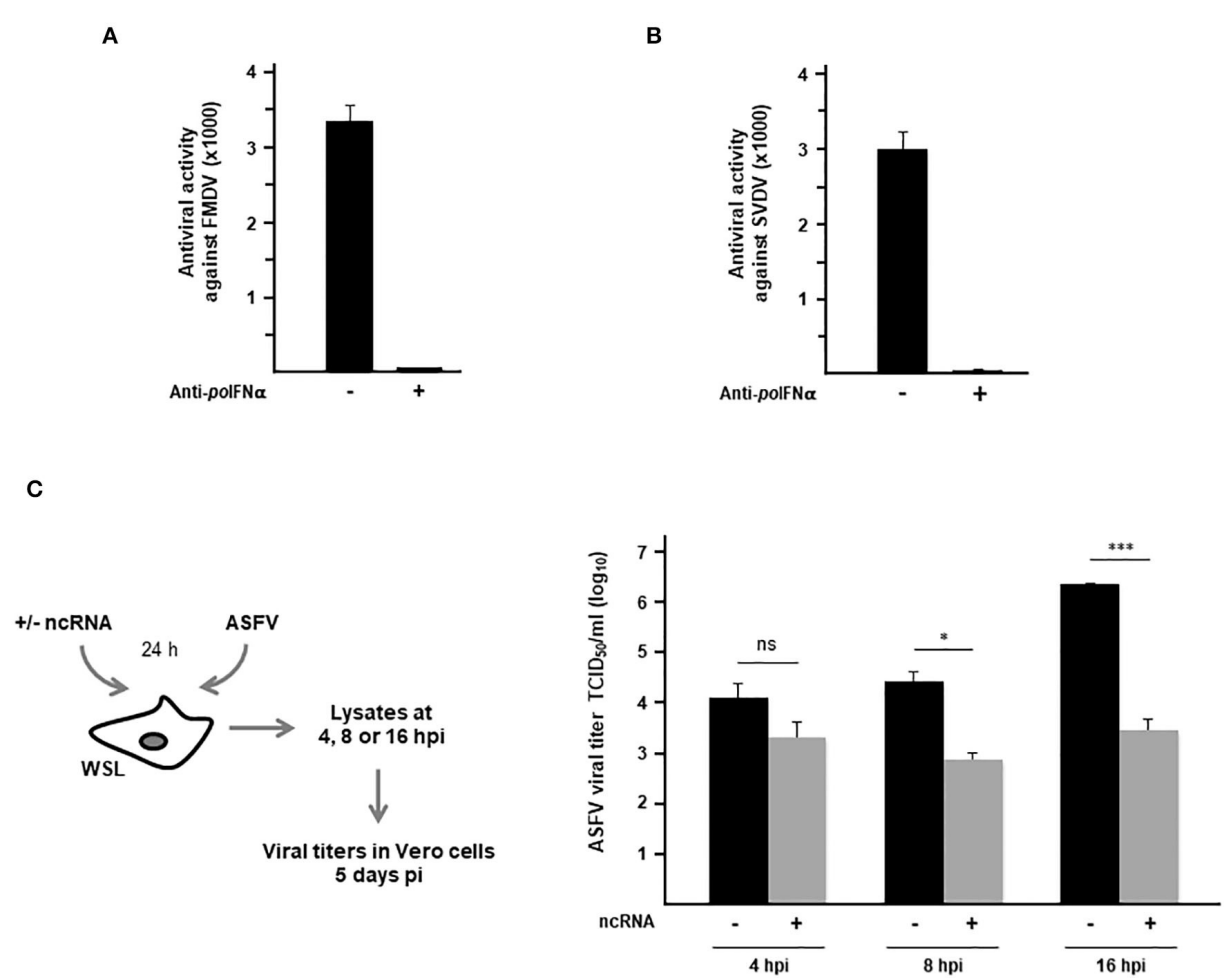
Antiviral activity against FMDV, SVDV and ASFV induced in WSL cells by transfection with the ncRNAs. **(A,B)** Supernatants from WSL cells transfected with IRES RNA for 24 h were assayed on fresh WSL monolayers against infection with FMDV **(A)** or on IBRS2 cells against infection with SVDV **(B)**. Where indicated, supernatants were incubated previously with antibodies against swine IFN-α. Antiviral activity was expressed as the reciprocal of the highest supernatant dilution needed to reduce the number of FMDV plaques by 50%. Data are mean ± SD of triplicates. **(C)** WSL cells were transfected with IRES transcripts or mock-transfected and 24 h later infected with ASFV at an MOI of 2. Lysates were collected at 4, 8 or 16 h after infection and viral titers were determined in Vero cells after 5 days of infection. Data are average of triplicates ± SD (*p < 0.05; ***p < 0.001; ns, not significant).

With the purpose of exploring the activity of the FMDV ncRNAs in wild boar cells against relevant viral pathogens affecting domestic pigs, antiviral activity assays were carried out against FMDV, SVDV and ASFV. When the paracrine antiviral activity in supernatants from WSL cells transfected with IRES transcripts was assayed against FMDV infection, very high levels of protection were observed with an average titer over 3,000 which was completely abrogated by previous treatment with anti-swine IFN-α antibodies (Figure 3A). Similarly, very high levels of antiviral activity against SVDV infection were observed (Figure 3B). In this case, supernatants of transfected WSL cells were assayed in swine kidney IBRS2 cells, as infection with SVDV induced a diffuse cell detachment but not a clear CPE in WSL cells (see Table 1). The role of wild boar in SVDV transmission is still controversial. While it is considered likely to be susceptible to SVDV infection, serological surveys suggest that wild boars do not serve as reservoir hosts in Europe (31). Next, we wanted to test whether ncRNA transfection in wild boar cells might have an inhibitory effect against infection with ASFV. Persistence of ASFV in wild boar in Eastern Europe remains a significant threat to domestic pig populations globally (32). Unlike VSV, FMDV, or SVDV, ASFV is a genetically complex double stranded DNA virus. As shown in Figure 3C, the differences between viral titers recovered from WSL cells that had been previously transfected with the IRES transcripts, compared with those in mock-transfected cells, increased over time as infection proceeded, and reaching statistical significance at 8 h post-infection. Remarkably, an 800-fold reduction in viral titers in IRES-transfected wild boar cells was observed at 16 h after infection with ASFV (Figure 3C).

WSL cells used in this study have a macrophage lineage origin. Though some of the viruses tested in WSL cells replicate mainly in epithelial cells, porcine cell lines developed from alveolar macrophages have been shown to be a valuable tool for viral pathogenesis and immune function studies, being susceptible to a wide variety of viruses including VSV and SVDV (33).

To conclude, the current study presents new data on the antiviral effect of the ncRNAs in wild boar cells, a wild animal host species for FMDV and many other pathogens affecting domestic pigs with a potential relevance in FMD epidemiology, especially considering the increasing population of the wild boar in Europe. Our results show a robust and broad range of antiviral activity against FMDV, other viruses causing vesicular disease in swine (VSV and SVDV) and ASFV, being the later, together with FMDV, a major concern in animal health worldwide. The possibility of implementing antiviral strategies in wild animals in contact with farm species during outbreaks is an interesting point for debate and further studies.

## Data Availability Statement

The original contributions presented in the study are included in the article/supplementary material, further inquiries can be directed to the corresponding author/s.

## Author Contributions

MR and MS designed the study. MS obtained funding and wrote the manuscript. MR and RH performed the experiments. All authors analyzed the data, revised, and approved the manuscript.

## Conflict of Interest

The authors declare that the research was conducted in the absence of any commercial or financial relationships that could be construed as a potential conflict of interest.
